# Three-Dimensional Bioprinted Autologous Minimally Manipulated Homologous Adipose Tissue for Skin Defects After Wide Excision of Skin Cancer Provides Early Wound Closure and Good Esthetic Patient Satisfaction

**DOI:** 10.3390/jcm14061795

**Published:** 2025-03-07

**Authors:** Reina Matsumura, Hajime Matsumura, Yuichiro Kawai, Jeehee Kim, Min-Chae Lee, Yeongseo Yu, Miki Fujii, Kazuki Shimada, Takako Komiya

**Affiliations:** 1 Department of Plastic and Reconstructive Surgery, Tokyo Medical University, Tokyo 160-0023, Japan; 2 Division of Plastic Surgery, Kumagaya Geka Hospital, Saitama 360-0023, Japan; 3 Division of Plastic Surgery, Fukaya Red Cross Hospital, Saitama 366-0052, Japan; 4 ROKIT HEALTHCARE, Seoul 08514, Republic of Korea

**Keywords:** adipose tissue, bioprinter, skin defect, skin cancer

## Abstract

**Background:** An autologous minimally manipulated homologous adipose tissue (AMHAT) fabricated using three-dimensional (3D) bioprinting has shown potential in the treatment of diabetic foot ulcers and other chronic wounds. **Methods:** This study assessed the efficacy of AMHAT fabricated using 3D bioprinting for treating skin defects after a wide excision of skin cancer lesions where primary closure was not feasible. A total of 10 consecutive patients were included. The wounds were photographed and analyzed using AI, and the fat tissue harvested from the lower abdomen was processed into an AMHAT patch using a 3D bioprinter. The wound area was measured weekly until complete wound closure was achieved. **Results:** The area of the skin defects ranged from 1.77–6.22 cm^2^, averaging 2.72 cm^2^. Complete closure was achieved in 3–5 (average, 4.2) weeks. The residual area decreased from 52% after 1 week to 3% after 4 weeks. The scar appearance was esthetically favorable, with one patient showing mild nostril asymmetry. Furthermore, patient-reported outcome evaluation using the Japanese version of SCAR-Q for postoperative scars showed a very high level of satisfaction. **Conclusions:** The 3D bioprinted AMHAT accelerates wound healing with minimal scarring, offering an important option for skin defects where esthetics are a priority.

## 1. Introduction

Skin cancer is one of the most common malignancies affecting the elderly, and its incidence is expected to increase significantly as the elderly population increases [1]. The principal treatment for non-melanoma skin cancer is complete resection with a safety margin.

Complete radical resection provides a safety margin of 2–5 mm for basal cell carcinoma (BCC) lesions measuring less than 2 cm and 4–6 mm for squamous cell carcinoma (SCC) [2,3]. Mohs micrographic surgery with intraoperative frozen sections to confirm the resection margins can also be performed, although this technique does not guarantee a complete cure without recurrence. After a wide excision with a safety margin, skin defects need to be reconstructed using a skin graft or flap if primary closure is not possible.

If residual disease is observed in the permanent pathological specimen, re-excision is necessary. The skin or flap graft must be removed, and the donor site area must be re-excised. Consequently, the resulting donor morbidity is very high. To avoid these situations, a two-stage reconstruction is recommended [2]. Recently, an artificial dermis (dermal regeneration template) has been used after a wide excision of skin cancer lesions. After confirming that the resected specimen has no residual disease, final wound closure with skin grafts is performed.

Even if the resection results in the exposure of cartilage and bone, the wound bed can be grafted after 2–3 weeks using an artificial dermis. However, the use of an artificial dermis has the disadvantage of a relatively long treatment period, as it takes 2–3 weeks before the graft can be placed on the artificial dermis and 1–2 weeks for the wound to stabilize after grafting. This prolonged period increases the risk of infection.

Because the face is a common area for the development of non-melanoma skin cancers [4], cosmetic appearance after treatment is also a significant issue for patients.

Skin graft of flaps leave a residual scar bordering the healthy skin, which is problematic in terms of reduced tissue malleability, length, color, thickness, and bulging.

Autologous fat grafting has been increasingly investigated in various fields. Mojallal et al. demonstrated that autologous fat grafting stimulates the neosynthesis of collagen fibers and improves angiogenesis and the thickness of the dermis and subcutaneous tissue [5]. Adipose tissue is rich in various growth factors (e.g., insulin-like growth factor, hepatocyte growth factor, transforming growth factor β1, and vascular endothelial growth factor), which in turn stimulate angiogenesis and epithelialization [5]. Adipose-derived stem cells are multipotent progenitor cells that can differentiate into different cell lineages such as fibroblasts, keratinocytes, and endothelial cells and are essential for tissue regeneration [6]. Several clinical trials have shown that growth factors, anti-inflammatory cytokines, angiogenic factors, and healing-related peptides in autologous fat grafts positively affect wound healing. In addition, studies have reported that the adipose tissue improves the healing rates of patients with diabetic foot ulcers (DFUs) [7]. Furthermore, autologous minimally manipulated homologous adipose tissue (AMHAT), fabricated using three-dimensional (3D) bioprinting technology, has been reported to be effective in the treatment of DFUs [8,9,10,11,12,13,14].

There are already 3D bioprinters that are capable of building human tissue with living cells, rather than creating inorganic prostheses [15]. Therefore, 3D printing technology has attracted significant attention, especially in plastic and reconstructive surgery and other fields, due to its incredible ability to create complex constructs with high precision to fit the defect or shape. Moreover, 3D bioprinted AMHAT can be engineered to match the exact size and depth of ulcer wounds using computer scanning technology [13]. As a result, it allows precise layering of cells, scaffolds, and biologic factors, enabling greater spatial control in tissue reconstruction [16].

The purpose of this study is to evaluate the efficacy of AMHAT fabricated using 3D bioprinting technology in wound management following wide excision of non-melanoma skin cancer lesions. Specifically, this study examines the speed of wound closure and esthetic appearance of full-thickness skin defects that cannot be sutured after skin cancer resection.

## 2. Materials and Methods

### 2.1. Study Protocol

In this single-arm, single-center pilot study of 10 consecutive patients, a 3D bioprinted AMHAT fabricated using the Dr. INVIVO device was used in patients diagnosed with non-melanoma skin cancer following a wide excision, depending on the pathologic type and location, where primary closure was not possible or not advisable.

The primary outcome measure was the period of complete epithelialization of the wound after treatment. Secondary outcome measures included the weekly wound area reduction rate and patient satisfaction with the scar at the site of the AMHAT applied at the final follow-up.

The study protocol was reviewed and approved by the internal review board of our hospital (study site), and informed consent was obtained from all participants.

Baseline demographic characteristics, including age, sex, comorbidities, and pathological diagnosis, were obtained from the medical records.

### 2.2. Surgical Procedure

The diagnosis of skin cancer was confirmed by a biopsy before surgery. During surgery, a complete excision was performed with appropriate margins, and the excised tissue was sent for a pathological diagnosis. The adipose tissue was harvested from the lower abdomen and used to manufacture the 3D bioprinted AMHAT.

The 3D bioprinted AMHAT was applied to the skin defect after the removal of the skin cancer lesion or after the partial wound was closed with primary closure. All surgical procedures were performed under local anesthesia.

### 2.3. Preparation of the AMHAT

**Figure 1:**
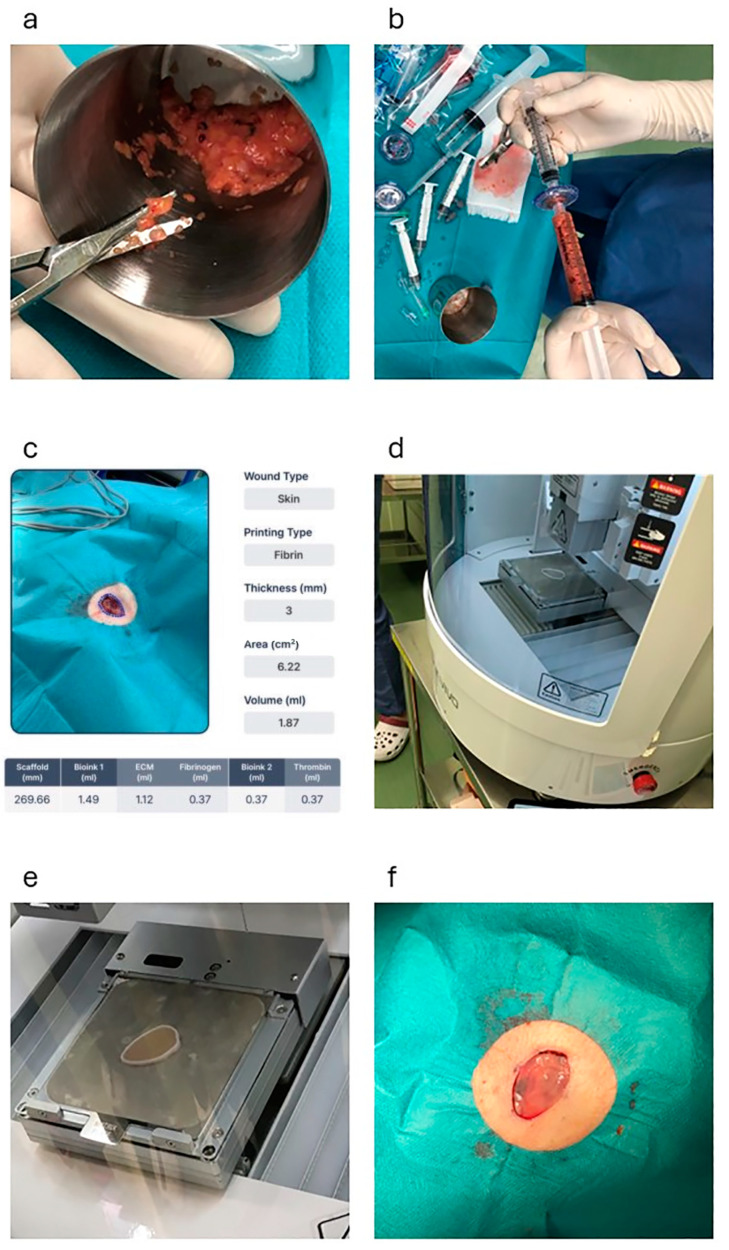
This figure shows the process of fabricating the 3D bioprinted AMHAT in case 1. (**a**) The adipose tissue is minced using scissors. (**b**) Micronization of the graft using a fat separator (Dr. INVIVO AI Regen KIT, ROKIT HEALTHCARE, Inc.). (**c**) Skin defects after skin cancer excision are scanned using an automated 3D modeling system (AiD Regen, ROKIT HEALTHCARE Inc.). (**d**,**e**) Creation of a scaffold by 3D printing using PCL. (**f**) Application of the 3D bioprinted AMHAT directly to the wound bed. AMHAT, autologous minimally manipulated homologous adipose tissue; 3D, three-dimensional; PCL, polycaprolactone.

On average, 10 mL of fat tissue was harvested from the lower abdomen of each patient. The collected adipose tissue was minced with scissors (Figure 1a) and immediately micronized using a fat separator device (Dr. INVIVO AI Regen KIT, ROKIT HEALTHCARE Inc., Seoul, Republic of Korea) to obtain the extracellular matrix (ECM) for fabricating the final patch (Figure 1b). The micronization process involved four meshes arranged in descending order by size (4000, 2400, 600, and 200 µm), through which the adipose tissue was progressively micronized. Sterile saline was added in a 1:2 ratio to wash and harvest the concentrated ECM, followed by gravity-based separation into three layers: free oil, ECM, and saline with blood. Only the ECM layer was collected, and the other layers were discarded and transferred to a new syringe.

### 2.4. Customizing the AMHAT Using a 3D Bioprinter

To fabricate a personalized ECM patch, the resection site was scanned using an automated 3D modeling system (AiD Regen, ROKIT HEALTHCARE Inc.) (Figure 1c). This software generates a highly accurate 3D model that replicates the precise dimensions and contours of a wound. The model was completed within 5 min and immediately uploaded to a 3D bioprinter (Dr. INVIVO, ROKIT HEALTHCARE Inc.) via an integrated application.

Upon receiving the model, the 3D bioprinter initiated the creation of a scaffold that served as a temporary mold for the ECM. This scaffold was printed using medical-grade polycaprolactone (PCL), which was engineered to precisely conform to the geometry of the wound (Figure 1d). After scaffold fabrication, bioprinting was employed to deposit the prepared ECM material onto the scaffold structure. To enhance the mechanical stability of the ECM, fibrin glue was applied to ensure secure adherence and solidify the ECM into a cohesive patch.

The entire process, from scaffold to ECM fabrication, was completed in approximately 30 min (Figure 1e). Once the ECM patch was fully solidified, the PCL scaffold was removed and the ECM patch (3D bioprinted AMHAT) was directly applied to the wound bed (Figure 1f). This application was followed by the placement of a primary silicone dressing (Mepitel ONE, Mölnlycke Healthcare, Mölndal, Sweden) and a secondary gauze dressing to ensure that the patch remained securely in place, thereby completing the procedure.

In this study, the ECM patch thickness was adjusted to 3–4 mm based on the depth of the wound site. Thickness optimization was achieved through precise parameter modifications using an automated 3D modeling system (AiD Regen, ROKIT HEALTHCARE Inc.), ensuring that the patch closely conformed to the affected area for effective tissue integration. This technology uses the boundary-guided point cloud processing method to filter data from images, accurately calculate the thickness and area, and convert them into a 3D model file [8].

### 2.5. Postsurgical Management

Mepitel ONE was removed 1 week after surgery, and, thereafter, the wound was treated daily by the patients themselves with an ointment containing gentamicin.

The wounds were then followed up weekly at the outpatient clinic until they were completely epithelialized. After complete wound closure, the patients were followed up for 6 months after skin cancer removal.

Digital photographs were recorded on the day of observation of the wound and its area was measured with ImageJ 1.54 [17] (NIH software, Bethesda, MD, USA) until wound closure. Wound area reduction was calculated as follows.

$$Wound\;Area\;Reduction\;(\%)=\left(\frac{Wound\;Area\;at\;each\;Week}{Baseline\;Wound\;Area\;\left(Week\;0\right)}\right)\times 100$$

This method normalizes the wound area measurements over time by expressing them as a percentage of the baseline (Week 0) wound area, allowing for a standardized comparison across different time points.

Digital photographs were also taken at the final follow-up visit to examine the cosmetic appearance of the wound and whether there was any recurrence of skin cancer.

### 2.6. Final Scar Appearance and Patient-Reported Outcome Measures Using SCAR-Q

At the final follow-up 6 months after surgery, the scar at the AMHAT transplant site was photographed and evaluated clinically. In addition to clinical evaluation, scarring was evaluated using the Japanese version of SCAR-Q [18], which is a patient-reported outcome measure. This evaluation consists of three items: appearance scale, symptom scale, and psychosocial impact. Each item is rated on a scale of 0 to 100, with higher scores indicating better outcomes, with a total score of 300.

## 3. Results

### 3.1. Patient Demographics

The demographic characteristics of patients are detailed in Table 1. There were an equal number of male and female patients, with a mean age of 73.6 (range, 61–84) years. There were five cases of BCC, four cases of SCC, and one case of SCC in situ. The areas of skin loss included the nasal area in five cases, the cheek in two cases, the cheek and nasal area in one case, and the clavicle region in one case. At baseline, the mean wound area was 2.72 cm^2^ (range, 0.5–6.22 cm^2^).

### 3.2. Wound Closure Process and Time to Complete Wound Closure

**Figure 2:**
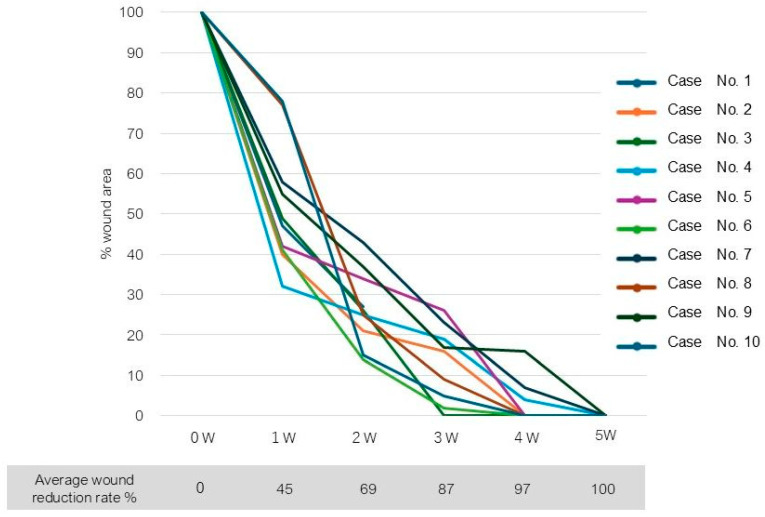
A Kaplan–Meier plot of wound area reduction.

Complete wound closure was achieved in 7 of 10 wounds by 4 weeks after surgery, and 3 wounds healed by 5 weeks. The mean time to wound closure was 4.2 weeks (Table 2). A Kaplan–Meier plot of wound area reduction is shown in Figure 2.

### 3.3. Final Scar Appearance and Patient Satisfaction

At the final follow-up 6 months after surgery, the scar was in an excellent condition. In case 7, in which complete closure took 5 weeks for the defect measuring more than 3 cm^2^ on the nose, asymmetry of the nostrils and mild contracture were observed; however, there was no desire for revision surgery.

The results of SCAR-Q were very good (265–300 points), with four patients scoring a maximum of 300 points. Photographs of the initial wounds, scars at the 6-month follow-up, and SCAR-Q scores are presented in Table 3.

### 3.4. Complications

Patients were monitored for adverse events, including local infection or graft loss, at each visit, and no adverse events were observed. No cancer recurrence was observed.

## 4. Discussion

If the defect following skin cancer removal is too large to be sutured, it is closed using a skin graft or flap. However, if wound closure is desired after confirming complete removal through the pathological examination of the excised tissue, a two-stage skin grafting procedure is performed using an artificial dermis.

Skin cancers often occur on the face and require color and texture matching of the skin during grafting.

Skin grafting from the periauricular area or supraclavicular fossa is considered a good choice; however, it leaves a scar on the exposed area.

When skin flaps are used for closure, the surgery and anesthesia are more invasive and are associated with complications [19].

In the present study, a 3D bioprinted AMHAT was used to treat skin defects after skin cancer excision where primary closure was not possible.

Upon the application of the 3D bioprinted AMHAT, which was composed of an adipose-derived ECM (microfragmented adipose tissue) and fabricated using AI and 3D printing technology, the growth factors, biological peptides, cytokines, and vascular components were delivered directly to the wound site. The bioengineered scaffold demonstrated precise edge fitting and maintained high cell viability, promoting the concentric alignment of regenerated cells and facilitating accelerated and effective wound healing (Figure 2).

Owing to the high wound healing ability of the AMHAT, it has been used to treat difficult-to-heal wounds, such as DFUs, with great success [8,9,10,11,12,13,14].

In the earliest cases, complete closure of the wound was achieved in 3 weeks, and, in several cases (6 of 10 cases), wound closure was completed within 4 weeks. The time taken for the wound to close was not significantly longer than that when a skin graft or flap was used to close the wound immediately after the cancer lesion was removed. It takes approximately 1 week for the skin graft or flap to take hold and another week or two for it to stabilize.

In this study, complete resection was achieved in all cases. However, if residual cancerous tissue is evident postoperatively, it is easy to re-excise and there is no need to discard the skin graft or flap; therefore, the sacrifice is very small compared with wound closure with a skin graft or flap.

Yun et al. [20] have evaluated the use of the AMHAT after skin cancer excision and reported that wound closure was statistically significantly earlier than that achieved with the application of a bilayer artificial dermis. However, although the total reconstructed area was larger in the present study, we achieved wound closure earlier. This may be attributed to the differences in the manner in which the fat patch was solidified. We used fibrin glue to solidify the fat tissue; however, Yan et al. solidified the fat patch by freezing the tissue. We believe that this difference affected the speed of wound closure. When the AMHAT is solidified using fibrin glue, the micronized adipose tissue is retained within the fibrin matrix, allowing for gradual absorption into the tissue over 2–3 days. In contrast, when AMHAT is applied in its frozen form, it dissolves within minutes due to body temperature. As a result, the composition of the AMHAT reaching the wound can differ depending on the solidification method. The combination of the AMHAT with fibrin glue creates an optimized wound-healing environment. Fibrin glue helps in tissue adhesion and may enhance the wound-healing properties of the AMHAT. These components simultaneously rapidly aid in cell proliferation and differentiation, resulting in a faster skin regeneration mechanism.

The adipose tissue from the abdomen was removed and used to create the 3D bioprinted AMHAT. Consequently, there was little morbidity at the donor site, and it did not cause any cosmetic problems. The adipose tissue may also be harvested by liposuction, in which case scarring of the donor site is close to none.

Despite the absence of a skin graft or flap, scar contracture was minimal, and the cosmetic result was highly satisfactory. This may be because AMHAT results in regeneration at the wound site rather than wound contraction [20].

In this study, we used the Japanese version of SCAR-Q, a patient-reported outcome measure, for evaluating postoperative scars. SCAR-Q is used to evaluate scars following craniosynostosis repair, breast implant reconstruction, and keloid treatment, and its effectiveness has been reported [18,21,22]. The results showed that patients were very satisfied with the appearance of their scars compared with those reported in previous studies. In particular, as 9 of 10 cases in this study involved exposed areas such as the face, this high level of patient satisfaction is very encouraging. In the psychological impact section of SCAR-Q, all participants gave full scores. This result may have been attributed to a sense of relief that the skin cancer had been completely removed or the use of new technology, and this may have led to a positive bias.

Until now, the AMHAT has often been used to treat difficult-to-heal wounds such as DFUs, owing to its high wound closure ability. In addition, the high esthetic quality of postoperative scars is a major benefit of using the AMHAT. In the future, we believe that this high quality of esthetics should be taken into account when considering this treatment option. Furthermore, in the future, it will be necessary to consider in detail the issues of the size of the skin defect and the location of the defect that can be esthetically satisfied with the 3D AMAHAT transplant.

This study has some limitations, including a lack of comparators with only one treatment group, patient recruitment from a single center, and a small sample size. A six-month follow-up period may not be sufficient to assess long-term scar quality, skin elasticity, functional outcomes, and the need for long-term follow-up of patients after healing. Additional comparative trials using randomized controlled trials and comparing artificial dermis, skin grafts, or untreated wounds should be conducted using this technique.

## 5. Conclusions

In this study, a 3D bioprinted AMHAT was used to treat skin defects after wide excision of skin cancer. Rapid wound closure was achieved within an average of 4.2 weeks after surgery, without the need for any additional procedures. The appearance of scars was esthetically very good, and patient satisfaction was very high, as evidenced by the very high SCAR-Q scores. These results suggest that the 3D bioprinted AMHAT accelerates wound healing with minimal scarring, offering an important option for large skin defects.

## Figures and Tables

**Table 1:** Demographic characteristics of patients.

Case No.	Age	Male/Female	Pathological	Location	Initial Wound Area (cm^2^)
			Diagnosis		
1	78	M	SCC	Clavicle	6.22
2	78	F	BCC	Nose	2.28
3	64	M	SCC	Nose	1.6
4	69	F	BCC	Nose	1.77
5	77	F	BCC	Nose	0.5
6	71	F	BCC	Nose~cheak	2.86
7	61	M	BCC	Nose	3.07
8	83	F	SCC	Cheak	2.92
9	71	M	SCC	Cheek	3.03
10	84	M	SCC in situ	Forehead	2.92
	Average	M:5			Average
	73.6	F:5			2.72

**Table 2:** Percentage of wound area reduction.

Case No.	Week 0	Week 1	Week 2	Week 3	Week 4	Week 5
1	6.22	2.95	1.67	ND	0	
2	2.28	0.92	0.47	0.37	0	
3	1.6	0.79	0.41	0		
4	1.77	0.57	0.45	0.34	0.07	0
5	0.5	0.21	0.17	0.13	0	
6	2.86	1.18	0.4	0.06	0	
7	3.07	1.79	1.31	0.72	0.21	0
8	2.92	2.25	0.74	0.26	0	
9	3.03	1.66	1.14	0.5	0.47	0
10	2.92	2.27	0.45	0.14	0	

**Table 3:** Initial wound, scar formulation at 6 months postsurgery, and SCAR-Q score of the patients.

Case No.	Wound After Cancer Excision	Scar at 6 months After Surgery	Appearance Scale Symptom Scale Psychosocial Impact	Scar-Q Total Score
1	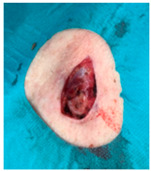	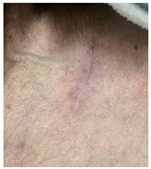	100 100 100	300
2	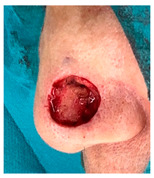	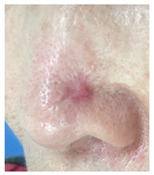	84 89 100	273
3	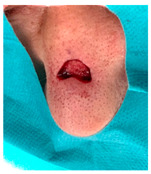	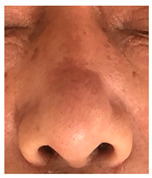	91 100 100	291
4	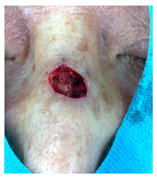	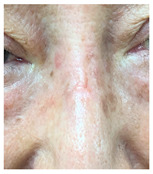	100 100 100	300
5	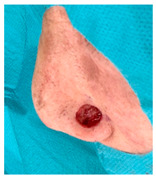	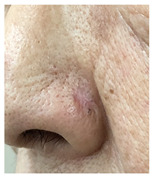	100 100 100	300
6	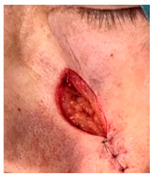	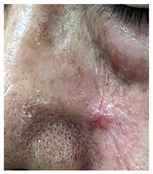	80 77 100	257
7	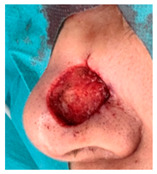	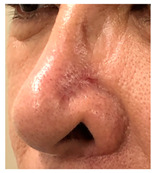	76 82 100	258
8	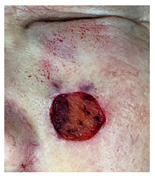	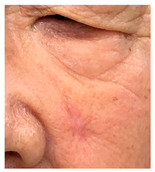	84 82 100	266
9	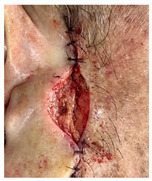	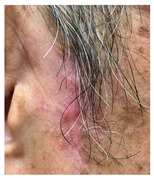	80 82 100	262
10	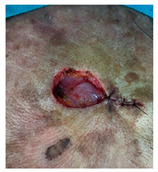	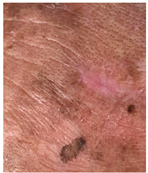	100 100 100	300

## Data Availability

All data analyzed in this study are available from the corresponding author upon request.

## Abbreviations

The following abbreviations are used in this manuscript:

AMHAT	autologous minimally manipulated homologous adipose tissue
3D	three-dimensional
AI	Artificial Intelligence
PCL	polycaprolactone
M	Male
F	Female
SCC	Squamous cell carcinoma
BCC	Basal cell carcinoma
ND	No data
